# *Boswellia frereana* suppresses HGF-mediated breast cancer cell invasion and migration through inhibition of c-Met signalling

**DOI:** 10.1186/s12967-018-1660-y

**Published:** 2018-10-12

**Authors:** Christian Parr, Ahmed Y. Ali

**Affiliations:** Connective Tissue Laboratories, Sir Martin Evans Building, School of Biosciences, Cardiff, UK

**Keywords:** *Boswellia frereana*, Breast cancer, Hepatocyte growth factor, c-Met, Invasion, Migration, Metastasis

## Abstract

**Background:**

Hepatocyte growth factor (HGF) plays a pivotal role in breast cancer cell motility, invasion and angiogenesis. These pro-metastatic events are triggered through HGF coupling and activation of the c-Met receptor. Reports have demonstrated that HGF/c-Met signalling plays an important part in breast cancer progression and that their expression is linked to poor patient outcome. In the present study, we investigated the anti-metastatic potential of an extract from traditional Somalian frankincense, *Boswellia frereana*, on human breast cancer cells. In addition, we also examined the effect of this *Boswellia frereana* extract (BFE) upon HGF-mediated stimulation of the c-Met receptor.

**Methods:**

Two triple negative human breast cancer cell lines, BT549 and MDA-MB-231, were utilised in the study to examine the effect of BFE on tumour cell proliferation, migration, matrix-adhesion, angiogenesis and invasion. Cell migration was investigated using a Cell IQ time-lapsed motion analysis system; while tumour cell–matrix adhesion, angiogenesis and invasion were assessed through Matrigel-based in vitro assays. Breast cancer cell growth and spheroid formation was examined through proliferation assay and 3D non-scaffold cell culture techniques. Western Blotting was employed to determine the phosphorylation status of the c-Met receptor tyrosine kinase following BFE treatment and subsequent HGF stimulation.

**Results:**

Following HGF treatment, the breast cancer cells displayed a significant increase in migration, matrix adhesion, vessel/tubule formation, invasion and c-Met activation. HGF did not appear to have any bearing on the proliferation rate or spheroid formation of these breast cancer cells. The addition of the BFE extract quenched the HGF-enhanced migratory, angiogenic and invasive potential of these cells. Further study revealed that BFE inhibited c-Met receptor tyrosine kinase phosphorylation within these breast cancer cells.

**Conclusions:**

Our findings reveal that BFE was able to significantly suppress the influence of HGF in breast cancer cell motility and invasion in vitro, through the ability of BFE to reduce HGF/c-Met signalling events. Therefore, these results indicate that BFE could play a novel role in the treatment of breast cancer.

## Background

Breast cancer is the most common cancer type in women, accounting for 29% of all new cancer diagnoses, and is the second most common form of cancer-related death in women worldwide [1, 2]. The presence of metastatic disease in breast cancer patients is the single most important factor affecting patient mortality. For this reason, attempts to control the metastatic spread of tumours remains a key target for the successful treatment of this disease [3]. However, the metastatic cascade is a complex multi-step process that is influenced by a host of factors, including the interaction between tumour cells and stromal-derived cytokines, which are key to the development and establishment of breast cancer metastases [4–7]. The stromal cells of mammary tumours produce a rich array of cytokines; one of the best documented is a cytokine known as hepatocyte growth factor (HGF).

Hepatocyte growth factor plays a crucial role in promoting breast tumour progression and metastasis. Upon complexing with its specific receptor c-Met, HGF evokes an array of biological responses within cancer cells, which subsequently lead to enhanced cell migration, matrix degradation, invasiveness and induction of angiogenesis. The significance of HGF activity in cancer development and progression has also been confirmed through clinical studies; where the degree of HGF and c-Met expression was found to correlate with disease progression and poor patient prognosis [8, 9]. The significance of the HGF/c-Met signalling in cancer has been extensively documented [10–15]. Collectively, these studies demonstrate that HGF and its receptor are strong therapeutic targets for cancer treatment and investigation of factors that govern the influence of HGF/c-Met coupling may lead to novel strategies to combat the metastatic spread of tumours.

Importantly, recent studies have also recognised the potential of the c-Met receptor as a target for treating the triple negative breast cancer (TNBC) subgroup of breast cancer patients [16, 17]. There are over one million new cases of breast cancer diagnosed globally each year, and of these approximately 15% are classed as triple-negative breast cancers as they are found to be lacking the oestrogen receptor, the progesterone receptor and the human epidermal growth factor receptor 2 [18–20]. Current successful hormone-based therapies directly target these three receptors, unfortunately patients with malignancies characterised as TNBC are associated with aggressive cancers with early metastatic spread and overall poor prognosis [21–23]. The poor outcome for patients with TNBC is due to the lack of a validated targeted therapy. Therefore, the recognition of a target genes, such as c-Met, is one of the most urgent current requirements for breast cancer treatment.

Traditional medicines are used by many people in the world today, generally because natural products are considered to be safe, inexpensive, and targeted towards a number of diseases. In most cases, however, neither the active components nor their mechanisms of action are well established. Frankincense is derived from the tree sap resin of the genus *Boswellia* and has been valued throughout the ages to have a wealth of healing properties. Scientific literature reports that a number of the *Boswellia* species, and the active component of *Boswellia* (boswellic acids), display anti-cancer properties through a capacity to reduce tumour growth and metastasis in a variety of established models [24–27]. Breast cancer studies have also demonstrated that *Boswellia serrata* and *Boswellia sacra* extracts have been developed to suppress the aggressive nature of breast cancer cells and their propensity to metastasise to secondary sites such as the brain [28, 29]. Presently, there are clinical trials underway examining the potential benefits of *B. serrata* treatment in the management of breast and colon cancer (ClinicalTrials.gov: NCT 03149081).

In this study we sought to investigate the anti-cancer properties of *Boswellia frereana*, a *Boswellia* species native to Somalia, on breast cancer cells. *Boswellia frereana* has been reported to act as an inhibitor of matrix metalloprotease 9 (MMP-9) activity during inflammation within an articular cartilage explant model [30]. HGF may also play a role in governing MMP-9 expression levels, as HGF antagonists have demonstrated the ability to downregulate MMP-9 activity in lung cancer cells [31]. This is the first study to assess the potential of BFE in cancer and we examined the effects of BFE on TNBC cell proliferation, migration, matrix-adhesion, invasion, angiogenesis and the activation/phosphorylation of the c-Met receptor under the influence of HGF. Here, we report that BFE suppresses HGF-enhanced cell migration, adhesion, vessel formation and invasion of breast cancer cells in vitro through inhibition of HGF/c-Met signalling and reduction of c-Met receptor phosphorylation.

## Methods

### Cells and materials

This study used the BT549 and MDA-MB-231 human TNBC cell lines, which were obtained from ATCC/LGC standard (Teddington, Middlesex, UK) and a human endothelial cell line (HECV) from Interlab Cell Line Collection (ICLC, Naples, Italy). Cells were routinely cultured with Dubecco’s modified Eagle medium (DMEM) supplemented with 10% fetal bovine serum, penicillin and streptomycin (Thermo Fisher Scientific, Paisley, UK). Breast cancer cells were passaged for less than 2 months before fresh cells were resuscitated from earlier cryogenically preserved stocks. Recombinant human hepatocyte growth factor was obtained from PeproTech (PeproTech house, London, UK) and was used at a final concentration of 10 ng/ml throughout the study unless otherwise stated. *Boswellia frereana* gum resin was purchased from Hargeisa, Somaliland, and successfully extracted as described previously [30]. Briefly, absolute ethanol was used to extract *B. frereana*, followed by removal of insoluble gum resin through filtration, and subsequent evaporation and solubilisation of the BFE in ethanol. The constituents of BFE were then analysed and verified through GC–MS.

### Cytotoxicity assay

BT549 and MDA-MB-231 breast cancer cells were seeded in a 96 well plate at a density of 5000 cells/well and incubated under routine conditions for 24 h. Following incubation, media was removed and replaced with media containing the appropriate concentration of BFE. The concentration of BFE added to the cells ranged from 500 μg/ml to 1 μg/ml. Breast cancer cells were then incubated for a further 72 h, followed by standard MTT assay assessment of cell viability. The sub-toxic concentration of BFE (highest concentration with 100% viability), and the LC50 values (concentration of BFE responsible for 50% cell death) were determined for each breast cancer cell line. The sub-toxic concentration of BFE in both cell lines was 10 ug/ml, and the LC50 values for BT549 and MDA-MB-231 were 27.2 μg/ml ± 2.3 and 29.8 μg/ml ± 2.1 respectively.

### Cell IQ cellular migration assay

Cellular migration assays were conducted using the self-contained Cell-IQ^®^ system (Chip-Man Technologies Ltd, Tampere, Finland) as described in detail previously [32]. Briefly, the Cell-IQ^®^ system is a fully integrated continuous live cell imaging and automated analysis platform which combines phase-contrast microscopy, environmental control (5% CO_2_ and maintained at 37 °C), with an on-board Analyser software package for the quantification of migration image data.

The migratory properties of the BT549 cells were assessed to determine the impact of HGF stimulation and BFE treatment on the aggressive motile nature of these breast cancer cells. Cells were seeded into a 24-well plate (Thermo Fisher Scientific, Paisley, UK) at 50,000 per well and incubated until confluence was reached. The monolayer was then scratched using a fine plastic pipette tip to create a wound of width approximately 300 µm. The media together with the floating cells were removed and replaced with media containing the treatment groups (Control ± HGF [10 ng/ml]; BFE [10 μg/ml] ± HGF [10 ng/ml]). The plate was immediately placed in the chamber of the Cell IQ unit and phase contrast images were automatically captured every 30 min for 6 h at pre‐programmed positions. Sequential images were analysed using the on-board Analyser software package.

The analysis software automatically calculated the mean width of the wound of each well at each time point. Total cell migration distance (µm) was calculated by deducting the calculated wound width from a specific time point, from the wound width at time 0, which then allowed the plotting of time course data. Whereas the migration rate (µm/h) was calculated by deducting the mean width of the wound from the mean width of the wound of the previous hour. This migration rate was calculated hourly for the duration of the experiment (6 h), and generated a mean migration rate for each treatment group for each time point. All experiments were conducted in triplicate.

### Breast cancer invasion assay

We quantified the invasive nature of the breast cancer cells using the standard invasion assay procedure as described previously [33]. Transwell chambers, equipped with a 6.5 mm diameter polycarbonate filter insert (pore size 8 μm)(Becton–Dickinson Labware, Oxford, UK), were pre-coated with 100 μg/insert of solubilised tissue basement membrane, Matrigel (Scientific Lab Supplies, Nottingham, UK). BT549 and MDA-MB-231 breast cancer cells were seeded at a density of 5000/insert and allowed to invade for 72 h in the presence of BFE (10 μg/ml) and/or HGF (10 ng/ml). Following incubation, cells that had invaded through the basement membrane were fixed (4% formaldehyde), and then stained with 0.5% crystal violet. For analysis, the cells were counted (10 fields/insert under ×40 magnification), to determine the mean number of invaded cancer cells for each treatment group.

### Cell–matrix adhesion assay

The cell–matrix adhesive properties of the BFE-treated cells to an artificial basement membrane were quantified using the in vitro Matrigel adhesion assay adapted from a previously described method [34]. Briefly, BT549 and MDA-MB-231 cells were seeded at a density of 10,000/well into a 96-well plate that had been previously coated with 5 µg of Matrigel artificial basement membrane. Cells were then routinely incubated for 30 min in the presence of the described treatments (Control ± HGF [10 ng/ml]; BFE [10 μg/ml] ± HGF [10 ng/ml]). Cells were allowed to adhere to the replica cell matrix, before two washing steps in PBS to remove non-adhered cells. Adherent cells were then fixed in 4% formaldehyde and stained with 0.5% crystal violet. Cells were counted in several fields/well under ×40 magnification to determine mean number of matrix-adhered cells per treatment group.

### 3D-culture spheroid formation

The development of spheroids through 3D-culture is more representative of tumour characteristics grown in vivo than traditional 2D-cultured cells. BT549 breast cancer cells will grow and form dense multicellular spheroids when cultured in the appropriate conditions. We used the ‘hanging-drop’ system which takes advantage of the fact that, in the absence of a surface to attach, BT549 cells will assemble into a 3D spheroid structure. Cells were added at 10,000 per well, to a critical final volume of 50 µl per well, in the various treatments groups (Control ± HGF [10 ng/ml]; BFE [10 μg/ml] ± HGF [10 ng/ml]). The aperture of each well in the plate (Perfecta3D hanging drop plate, Sigma-Aldrich, UK), is designed so that when the cells are carefully dispensed into the well, a hanging droplet is formed that is small enough to remain suspended through surface tension. The cells were then cultured under routine conditions for 4 days to allow the cells to grow and develop into spheroids within the hanging droplet. Images were collected and the mean size/area of each spheroid was calculated for each treatment group.

### Proliferation assay

BT549 and MDA-MB-231 breast cancer cells were seeded in a 96 well plate at a density of 5000 cells/well and incubated under routine conditions for 24 h. Following incubation, media was removed and replaced with media containing the appropriate treatment (Control ± HGF [10 ng/ml]; BFE [10 μg/ml] ± HGF [10 ng/ml]), and then incubated for a further 72 h prior to standard MTT assay assessment of viable cell number.

### Tubule formation assay

Human umbilical cord endothelial cells (HECV) were plated into a 24‐well plate pre‐coated with Matrigel (diluted 1:1 with serum‐free media), at a seeding density of 20,000/well. Treatments were added (Control ± HGF [10 ng/ml]; BFE [10 μg/ml] ± HGF [10 ng/ml]) and tubules were allowed to form over a 24 h incubation period. Images were captured to analyse the degree of tube/vessel formation as described previously [35].

### SDS PAGE and Western blotting

BT549 breast cancer cells were seeded into a 6 well plate and allowed to grow until 70–80% confluence, whereupon cells were subjected to overnight serum starvation in a serum free culture medium. The appropriate treatments were then added to the cells (Control ± HGF [10 ng/ml]; BFE [10 μg/ml] ± HGF [10 ng/ml]), for a 60 min incubation period. This was followed by standard cell lysis, SDS-PAGE and Western Blot techniques. The c-Met phosphorylation status and c-Met total protein levels were assessed using the Phospho-Met (Tyr1234/1235) and Met (25H2) Mouse mAb antibodies respectively. All antibodies were purchased from Cell Signalling Technology unless otherwise stated (Cell Signalling Technology, Leiden, Netherlands). STAT-3 and JAK-2 activity was examined with the Phospho-Stat3 (Tyr705) and the Phospho-Jak2 (Tyr1007/1008) antibodies. B-actin expression levels were used as an internal control (Santa-Cruz Biotechnologies, California, USA). Protein expression was assessed and quantified using Image J analysis software.

### Gelatinase zymography

Gelatinase zymography was performed to assess the expression levels of both the inactive pro-form and biologically active form of MMP-2 and MMP-9 that are produced by the BT549 and MDA-MB-231 cell lines. Breast cancer cells were seeded into a 6 well plate and allowed to grow until 50% confluence, whereupon cells the appropriate treatments were then added to the cells (Control ± HGF [10 ng/ml]; BFE [10 μg/ml] ± HGF [10 ng/ml]), and incubated for an additional 4 days (80–90% confluence). Cell supernatant was then collected from each treatment group and samples were processed and prepared for standard gelatinase zymography as described previously [30].

### Statistical analysis

The results were assessed using one-way ANOVA test with post hoc Tukey HSD, and non-paired two-sided Student’s t-test. A *p*-value < 0.05 was defined as statistically significant.

## Results

### *Boswellia frereana* extract suppressed the migratory potential of HGF-induced breast cancer cells

**Fig. 1:**
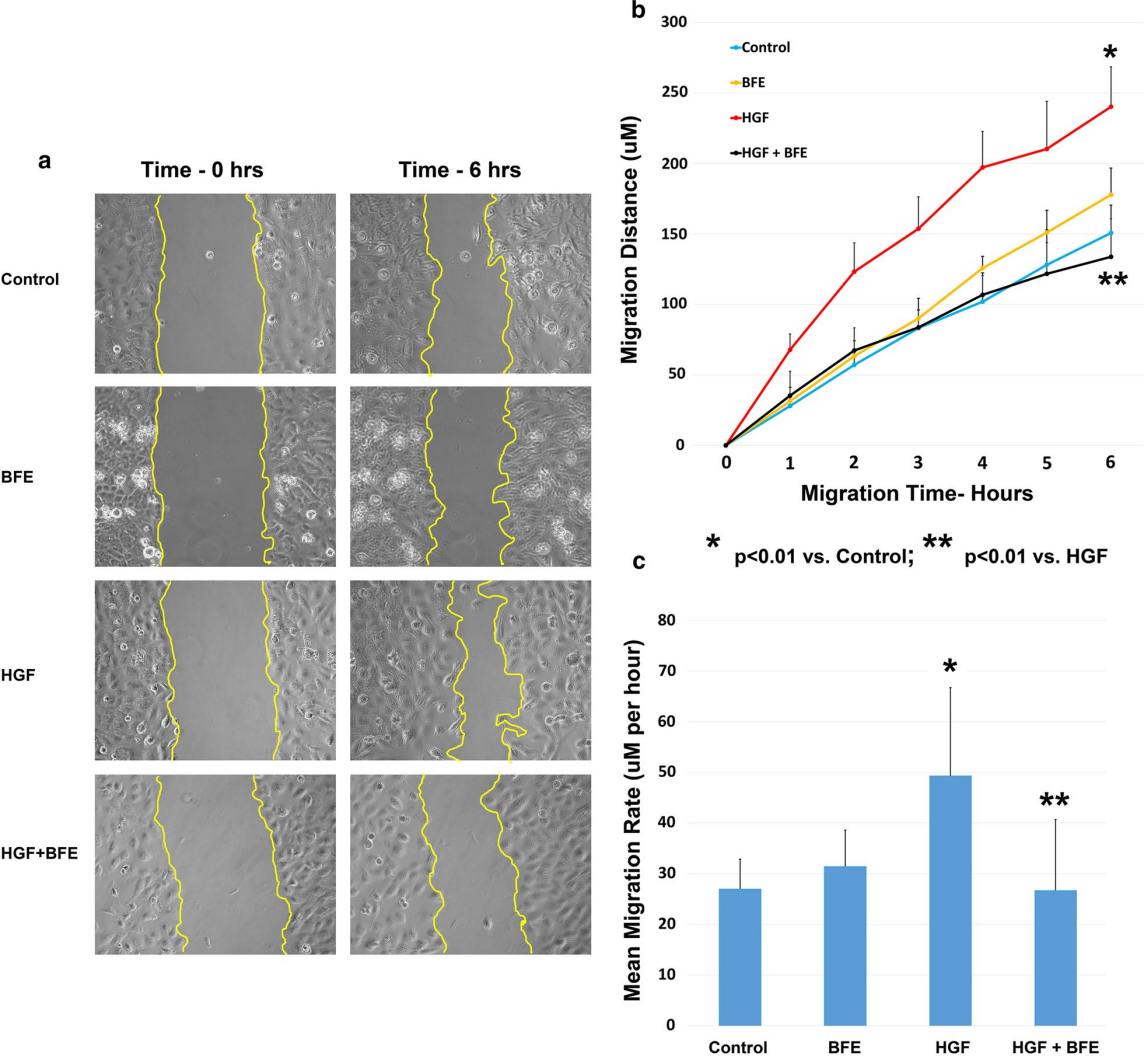
BFE suppressed the HGF-induced motile properties of breast cancer cells: **a** images taken from the BT549 wounding model. The leading edge of the wound has been high-lighted yellow. The left hand panel of shows the treatments groups at time zero, and the right hand panel reveal the degree of wound closure/cell migration for each treatment group following 6 h incubation post wounding. During the course of the experiment HGF-treated cells have migrated at a faster rate compared to the other groups and have almost closed the wound, an effect negated by the addition of BFE. **b** The on-board Cell-IQ analyser software has calculated the mean width of the wound (µM), and we have used the data to calculate the migration distance over the 6 h. HGF significantly enhanced the total migration distance of the cells; whereas HGF in combination with BFE showed no difference to the control. **c** The migratory rate (µM/h) was also quantified and we reveal that again HGF increased the rate of the migration, while addition of BFE suppressed the migratory potential of the HGF-stimulated cells

The Cell-IQ^®^ system with an on-board Analyser software package was utilised for the measurement and quantification of the migratory properties of the BT549 cells. We determined the impact of HGF stimulation and BFE treatment on the aggressive motile nature of these breast cancer cells every 60 min over a 6 h period. The degree of migration could be observed through comparison of images collected at time zero of the experiment and 6 h post-wounding (Fig. 1a). The HGF treatment (10 ng/ml) evoked a strong migratory response in the cells, demonstrated by the enhanced level of cell movement/wound closure when compared to the control cells. The addition of BFE alone (10 μg/ml) did not reveal any obvious differences to the untreated cells, however the additional of BFE in combination with HGF revealed a dramatic reduction in the migration of the breast cancer cells after 6 h when compared to the HGF treatment group.

This system also facilitated the full quantification of the breast cancer cell migratory distance (µM) throughout the course of the experiment (Fig. 1b). There was no significant difference (*p *= 0.32), between the untreated control group of BT549 breast cancer cells and the BFE-treated cells, which migrated a mean total distance of 150.8 ± 19.7 µM and 177.8 ± 19 µM, respectively over the 6 h period. The HGF-stimulated treatment group revealed a dramatic increase (*p *= 0.0025) in total cell migration distance (240.3 ± 28.3 µM) when compared to the control group. However, this HGF-induced effect on total cell migration was significantly suppressed (*p *= 0.0056) through the addition of BFE in the HGF + BFE treatment group (133.8 ± 27 µM).

Furthermore, the actual migration rate of the leading edge of the cells was also assessed every 60 min for the duration of the experiment (Fig. 1c). The mean migration rate per hour was determined for each treatment group and we report that the presence of HGF significantly (*p *= 0.0035) enhanced the migration rate (49.31 µM/h) of these breast cancer cells compared to the control group (27.01 µM/h). Importantly, this elevation in motility by HGF was quenched (*p *= 0.0013) through the addition of BFE, as demonstrated by comparison with the HGF + BFE group (26.69 µM/h). There was no significant difference (*p *= 0.123) between the control group and the BFE alone treatment group (31.44 µM/h).

### *Boswellia frereana* extract had a significant impact on breast cancer cell invasion and cell–matrix adhesion

**Fig. 2:**
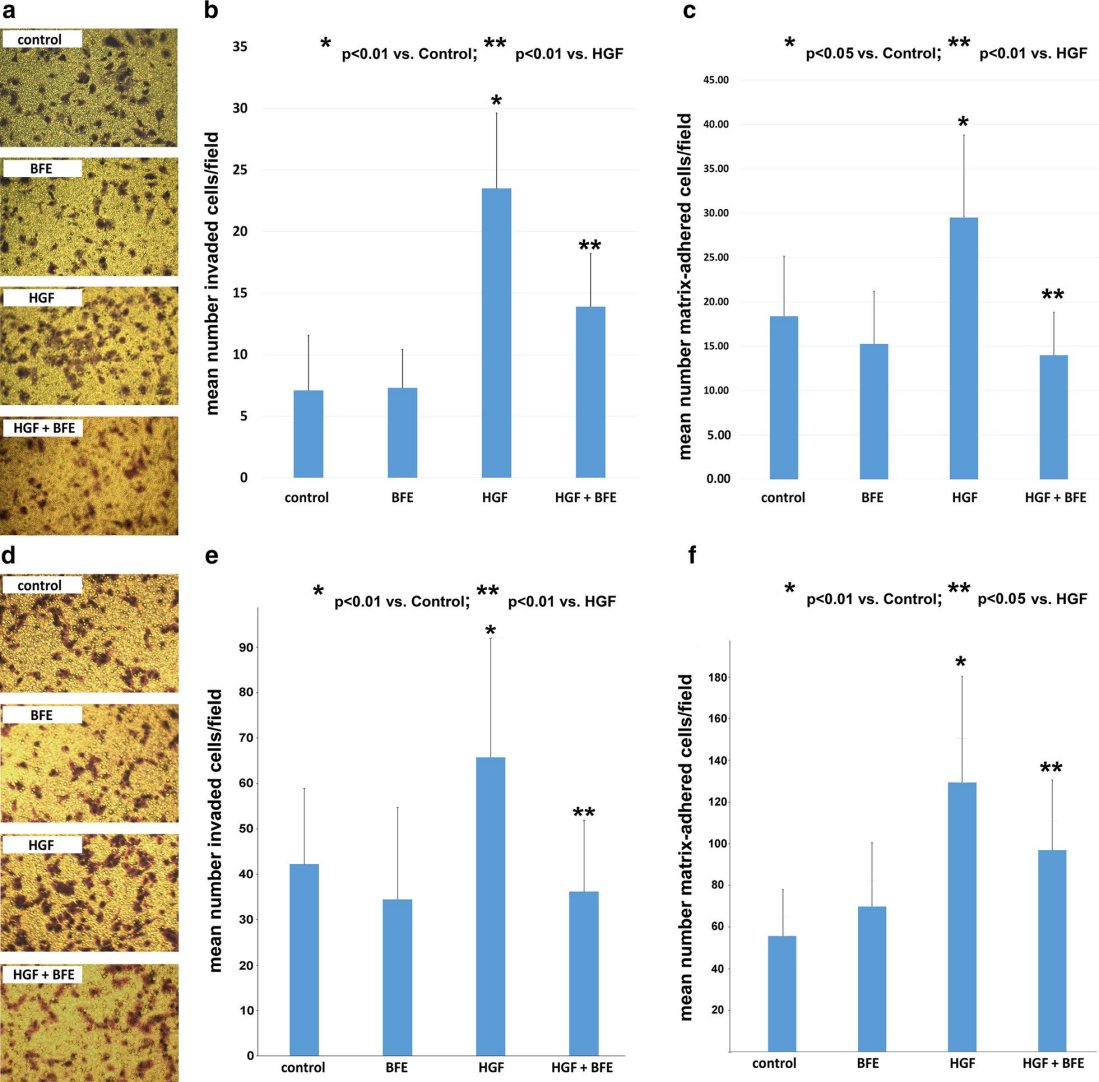
HGF-stimulated breast cancer cell invasion and cell–matrix adhesion was inhibited through BFE treatment: **a** BT549 cell line; **d** MDA-MB-231 cell line—Images taken following a 48 h incubation period using the breast cancer cell invasion model. The enhanced degree of invasion through HGF stimulation was observed through the higher level of successfully invaded breast cancer cells when compared to the control group. BFE was able to suppress this effect when incubated with HGF, and importantly addition of BFE alone had no bearing on the degree of breast cancer cell invasion. **b** BT549 cell line; **e** MDA-MB-231 cell line—the number of cells that successfully invaded were quantified and results plotted to reveal the statistically significant increase in cell invasion following HGF treatment, which was significantly inhibited through BFE treatment of breast cancer cells. **c** BT549 cell line; **f** MDA-MB-231 cell line—the number of breast cancer cells that formed a strong attachment to a replica matrix during the allotted time was quantified and the data reveals that HGF significantly enhanced cell–matrix adhesion and that the edition of BFE was able to quench the degree of attachment by these cells

Breast cancer cell degradation and invasion through the tissues are key events in the metastatic cascade. We used an in vitro replica extracellular matrix invasion model to assess and quantify the aggressive nature of the BT549 (Fig. 2a–c) and MDA-MB-231 (Fig. 2d–e) TNBC cell lines. Breast cancer cells were cultured with various treatments for 72 h, following which the degree of invasion was assessed in both cell lines (Fig. 2a, d) and quantified to reveal the effect of BFE on HGF-stimulated cells (Fig. 2b, e). We report that these TNBC breast cancer cell lines are highly invasive and cells spontaneously invade through the artificial matrix as demonstrated by the control groups (BT549: 7.1 ± 4.4; MDA-MB-231: 42.3 ± 16.6). The pro-metastatic influence of HGF on these cells was further demonstrated, as the breast cancer cells that were cultured in the presence of HGF showed a dramatic increase in the number of cells invaded (BT549: 23.5 ± 6.1, p = 0.001; MDA-MB-231: 65.8 ± 26.2, p = 0.005). Importantly, we reveal that when BFE was added to the HGF culture environment the level of invasion was significantly reduced (BT549: 13.9 ± 4.3, p = 0.001; MDA-MB-231: 36.2 ± 17.7, p = 0.001). We also report that BT549 cell treatment with BFE alone had no detrimental effect on cell number and did not reveal any statistical difference when compared against the control group (BT549: 7.3 ± 3.1, p = 0.90; MDA-MB-231: 34.5 ± 20.2, p = 0.25).

Cell–matrix adhesion is a complex process involving cell integrin mediation of the many cell–matrix interactions which contribute to cancer progression and facilitates tumour cell matrix degradation and invasion. HGF promotes cancer cell adhesion to components of the extracellular matrix (ECM). The ability of BFE to limit HGF-induced matrix adherence of the TNBC cancer cells was demonstrated through the attachment assay (Fig. 2c, f). The results show that the control group of untreated breast cancer cells strongly adhered to the replica basement membrane following 30 min incubation (BT549: 18.38 ± 6.78, MDA-MB-231: 56 ± 22.6), however stimulation with HGF revealed a dramatic increase in cell attachment (BT549: 29.5 ± 9.3, p = 0.016; MDA-MB-231: 129.9 ± 51.1, p = 0.001). Crucially, the influence of HGF could be negated through addition of BFE during the culture process. BFE significantly inhibited the degree of HGF-enhanced cell–matrix adhesion (BT549: 14.0 ± 4.84, p = 0.001; MDA-MB-231: 97.2 ± 34, p = 0.03). Whereas, the inclusion of BFE alone had no bearing on cell–matrix adhesion when compared to the control group (BT549: 15.25 ± 5.95, p = 0.78; MDA-MB-231: 70.1 ± 30.9, p = 0.13). Once again, these results suggest that the presence of BFE was able to limit the pro-metastatic influence of HGF on breast cancer cells.

### *Boswellia frereana* extract did not affect breast cancer cell proliferation or development of 3D cultured spheroids

**Fig. 3:**
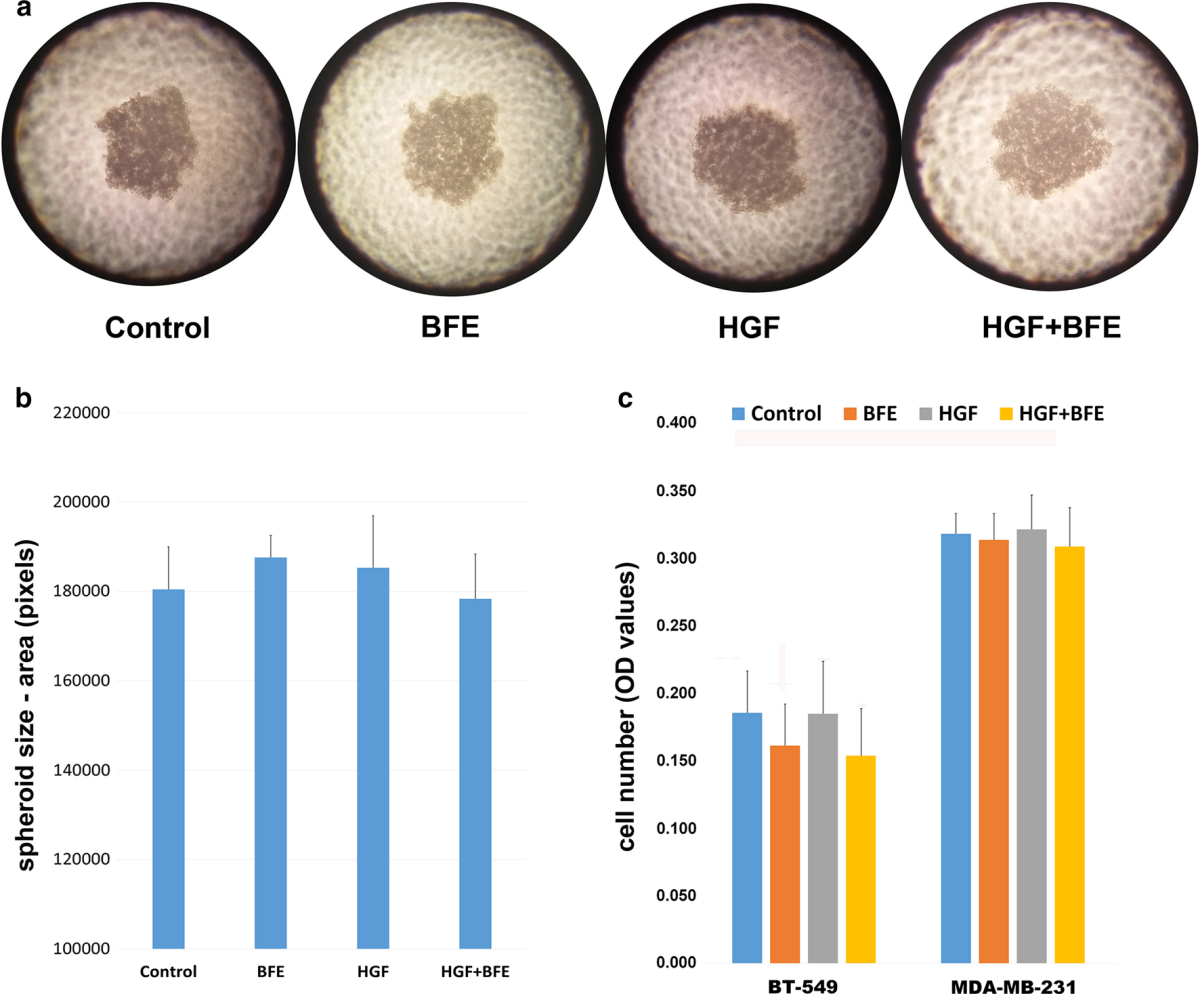
BFE and HGF had no effect on breast cancer cell proliferation and the formation of spheroids: **a** images taken of the degree of spheroid formation within the hanging droplet following 4 days incubation. The BT549 breast cancer cells successfully formed spheroid structures, however there was no obvious differences between the different treatment groups. **b** The spheroid size was calculated for each group and the results plotted. These results report that HGF and BFE have no impact on the ability of these breast cancer cells to form spheroid structures. **c** The results from a separate BT549 and MDA-MB-231 proliferation studies also confirm that neither HGF nor BFE have any bearing on the proliferation rate of these breast cancer cell lines

Using the hanging drop 3D culture system we investigated the potential of the breast cancer cells to grow and form spheroid structures when cultured under the influence of HGF. Following 4 days culture within the various treatment groups, the development of spheroids was assessed microscopically (Fig. 3a), and we report that the BT549 breast cancer cells successfully developed into spheroid structures, whereas the MDA-MB-231 breast cancer cells did not. However, further measurement and analysis of BT549 spheroid formation (Fig. 3b) revealed that the addition of HGF and or BFE had no bearing on spheroid growth and development.

To exclude the possibility that the above-mentioned inhibitory effects of BFE on HGF-induced cell migration, adhesion and invasion could be attributed to a change in the proliferation rate and growth of the BT549 or MDA-MB-231 cells, we employed a standard MTT assay to examine viable cell number after 72 h culture within the appropriate treatments. Following incubation, our results (Fig. 3c) indicated that there were no significant difference in breast cancer cell number between the various treatment groups with HGF, BFE or a combination of the two.

### HGF-enhanced vessel formation was inhibited through the addition of *B. frereana* extract

**Fig. 4:**
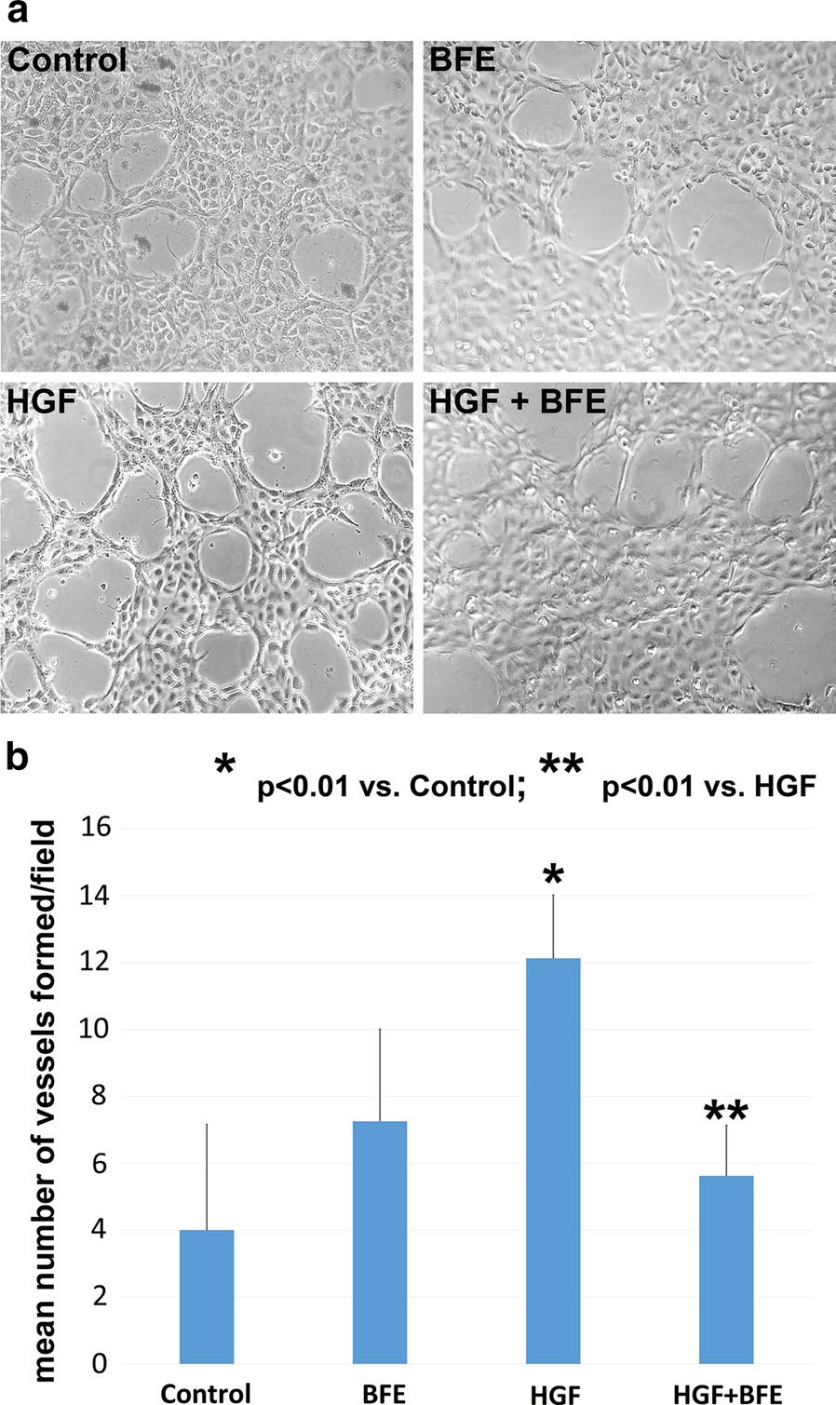
BFE was able to suppress the pro-angiogenic effect of HGF on human endothelial cells: **a** images taken demonstrating the degree of tubulogenesis/vessel formation by the HECV human endothelial cell line. Using the matrigel-based in vitro angiogenesis model, we demonstrate that stimulation of these cells with HGF resulted in an increased level of vessel formation. Importantly, the addition of BFE in combination with HGF was able to suppress the degree of tubulogenesis. **b** Images were analysed and the level of vessel formation was quantified. Results confirm that HGF statistically enhanced the level of angiogenesis of these HECV cells and that treatment of these cells with BFE reduced the degree of vessel formation significantly

Neovascularisation, a vital part of breast tumour growth and progression, can be assessed in vitro using an angiogenesis/tubulogenesis assay. This technique is based on the ability of endothelial cells (HECV) to form tube/vessel-like structures when cultured upon a Matrigel extracellular matrix under the influence of pro-angiogenic factors such as HGF. We examined the ability of HGF to stimulate vessel formation and the capacity of BFE to limit this effect (Fig. 4a). The degree of vessel formation was determined (Fig. 4b), and we report that the HECV cells formed micro vessels in the absence of a stimulus (4 ± 3.1); however the cells treated with HGF displayed a significant increase in the level of vessel formation (12.16 ± 1.89; p = 0.001). We reveal that BFE, incubated with HGF, significantly reduced the effect HGF evokes on HECV vessel formation (5.63 ± 1.51; p = 0.001). The addition of BFE alone did not display any significant influence on vessel formation when compared to the control group (7.25 ± 2.75; p = 0.18).

### *Boswellia frereana* extract reduced HGF-mediated breast cancer aggressiveness through suppression of tyrosine phosphorylation of the c-Met receptor

**Fig. 5:**
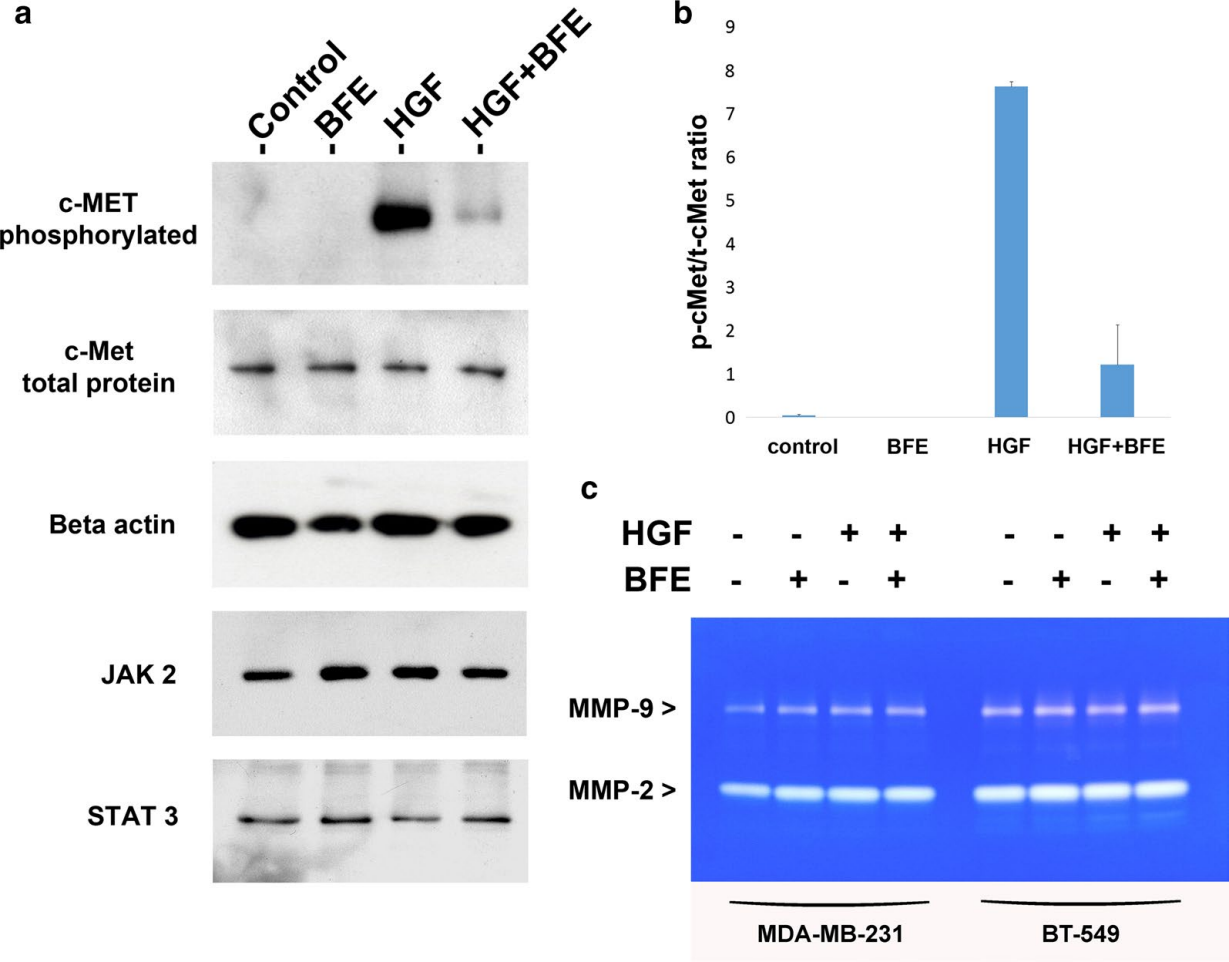
BFE quenched the HGF-enhanced breast cancer aggressiveness through inhibition of c-Met receptor phosphorylation: **a** the images show the phosphorylation status of c-Met, Stat3 and Jak2; and also the total protein levels of the c-Met receptor and the beta-actin control within the BT549 cell line following SDS-PAGE and Western blotting procedures. The different treatment groups displayed no significant differences when comparing the total protein levels for c-Met and the beta-actin control. Importantly, we show that HGF-stimulation of these cells resulted in a significant increase in activation and phosphorylation of the c-Met receptor. This HGF-enhanced phosphorylation was quenched through the co-incubation of BFE. **b** The ratio between phosphorylated c-Met and total c-Met was calculated to and plotted to demonstrate that the addition of BFE, rather than aberrant protein levels, was responsible for the shift in receptor phosphorylation status. **c** Gelatinase Zymography revealed no significant differences in MMP2/9 expression levels within the breast cancer cells following HGF/BFE treatments

The events associated with HGF-induced breast cancer cell metastasis are triggered through HGF coupling with c-Met and the subsequent activation of signalling pathways that promote tumour progression. This study investigated the phosphorylational status of the c-Met receptor within the TNBC cells, following HGF stimulation, to determine if BFE inhibited HGF action through suppression of c-Met receptor phosphorylation. In addition, this study also assessed the activity and expression levels of a number of factors involved in epithelial-mesenchymal transition (EMT), including STAT-3, JAK-2, MMP-9 and MMP-2. Following overnight serum deprivation, in the absence of HGF, we report that the BT549 cells displayed no c-Met activation (Fig. 5a). However, following HGF treatment for 60 min the cells revealed a high degree of c-Met phosphorylation. Crucially, we report that incubation of BFE with HGF resulted in a significant reduction in c-Met activation (Fig. 5a). There were no differences in total c-Met protein levels expressed between the treatment groups; therefore, upon calculating the ratio of phosphorylated c-Met to total c-Met (Fig. 5b), we demonstrate that the reduction of HGF-induced phosphorylation was due to the treatment of these breast cancer cells with BFE rather than aberrant protein levels. We also report that the levels of STAT-3 and JAK-2 were found to be constitutively active in the BT549 breast cancer cell line and treatment with HGF and/or BFE had no bearing on the phosphorylational status (Fig. 5a). Zymography also revealed that both MMP2 and MMP9 were released as inactive pro-forms by both TNBC cell lines (Fig. 5c). However, no differences were observed between MMP expression levels following HGF/BFE treatment.

## Discussion

In the present study, we have demonstrated for the first time that an extract from *B. frereana* resin was able to suppress the influence of HGF on the BT549 and MDA-MB-231 TNBC cell lines in vitro. Upon HGF stimulation, these breast cancer cells revealed a dramatic and significant increase in motility, cell–matrix adhesion and invasiveness; this increase was governed through HGF-Met coupling and subsequent activation of the c-Met receptor. HGF stimulation did not appear to have any impact on the proliferation rate or the growth and formation of 3D spheroids within these breast cancer cells. HGF also increased the degree of tubulogenesis/vessel formation when added to a human endothelial cell line. However, the addition of the *B. frereana* extract quenched all these HGF-induced pro-metastatic events in vitro without affecting the growth of these cells. Further study revealed that the BFE inhibited these HGF-enhanced effects through suppression of c-Met receptor tyrosine kinase phosphorylation (pY1234/5) within these breast cancer cells.

This study also assessed a number factors that are up/down-regulated during the process of epithelial-mesenchymal transition (EMT). EMT occurs through the disassembly of epithelial cell–cell contacts and reorganisation of ECM to facilitate cell migration, invasion and metastasis. HGF is believed to act as an initiation signal for the onset of EMT, which results in the upregulation of effector molecules, such as MMP-2 and MMP-9, and activation of STAT-3 to aid EMT and the metastatic dissemination of breast cancer cells [4, 36, 37]. Our results suggested that treatment with HGF and/or BFE did not appear to have any bearing on the activity or expression levels of these factors within these breast cancer cell lines.

Breast cancer cell invasion and establishment of metastasis are devastating events for patients with cancer. Patients classified with TNBC have a type of cancer that is characterised by the aggressive invasive clinical behaviour of the tumour which has a propensity to metastasise early and establish secondary tumours at additional sites. Furthermore, TNBC patients have limited targeted treatment options due to the fact that they do not express any of the three receptors directly targeted by the current successful therapies. For these reasons TNBC patients are generally considered to have a poor prognosis, thus emphasising the importance of recognition and validation of novel targets for the inhibition of metastasis in TNBC.

Hepatocyte growth factor and its partner c-Met play a definitive role in the development and progression of breast cancers, as well as being implicated in particularly invasive and metastatic cancers. HGF/c-Met are logical therapeutic targets in the treatment of TNBC. Strategies targeting HGF/c-Met activity have warranted further investigation for their potential in combating the metastatic spread of tumours. In recent years, the blockade of HGF-Met signalling has become one such strategy to inhibit tumour invasion and metastasis [10, 38]. Clinical studies have demonstrated the potential of c-Met inhibitors as a novel form of targeted therapy, as an investigation into primary breast cancer patients revealed that elevated phosphorylated c-Met expression (pY1234/5) was found to be associated with TNBC patients [39]. Several reports review the clinical trials that are presently evaluating the benefit of anti-c-Met and anti-HGF monoclonal antibodies and tyrosine kinase inhibitors in a variety of cancer types including TNBC [9, 16, 40]. The clinical relevance of c-Met inhibitors in breast cancer is under investigation with phase II clinical trials evaluating the potential of tivantinib in patients with recurrent or metastatic TNBC [41] and in a randomized phase II study assessing the safety and efficacy of onartuzumab and/or bevacizumab in combination with paclitaxel in patients with metastatic TNBC [42], where the aim is to identify patients who would benefit from c-Met targeted therapy. Further progress will lead to the application of these advances in the generation of future therapies to prevent the spread of cancer in TNBC patients. To date, the most promising approach for disrupting HGF/c-Met signalling appears to be small molecular inhibitors that target the intracellular kinase domain [43].

We demonstrate that BFE had a significant and direct effect on HGF-induced migration, adhesion, invasion and tubulogenesis through the inhibition of HGF/c-Met signalling and reduction in c-Met tyrosine kinase phosphorylation. This shift between HGF-induced c-Met activation and inhibition of c-Met signalling by BFE, could be a pivotal step controlling the metastatic influence of HGF, thus limiting breast tumour progression. The binding of HGF to c-Met and the subsequent activation of the intracellular domain occurs through a process of phosphorylation of the two tyrosine residues in the catalytical regions Y1234 and Y1235, followed by phosphorylation of two docking tyrosines (Y1349 and Y1356) [44]. This results in the recruitment of adaptor proteins which facilitate the binding to multiple substrates [45], which in turn leads to the activation of a variety of downstream intracellular signalling pathways (such as PI3K-AKT, MAPK, STAT and NF-κB), which are responsible for driving cell proliferation, invasion, migration, angiogenesis and cell survival [46, 47]. The triggering of downstream signalling events upon c-Met activation appears to be cell or context specific [16].

One study reveals that a specific c-Met tyrosine kinase activation pathway correlates with high risk patients who will go on to develop an aggressive form of the disease; this also suggests that the determination of this c-Met activation signature pathway may be used as a clinical tool to identify and predict patient response for future personalised anti-Met therapies [48]. The use of such clinical tools will play a key role in the management of breast cancer and may be used in the process of stratifying patients accordingly. It is important to identify those subgroups of patients most likely to benefit from anti-Met therapies, such as those with TNBC or further sub-classifications of TNBC. The outcomes of current and future c-Met clinical trials are eagerly anticipated. However, Ho-Yen et al. [10], reveal that such issues as resistance and c-Met receptor cross-talk with other receptor tyrosine kinases need to be addressed if treatment efficacy is to reach its full potential.

## Conclusions

Our findings reveal that *B. frereana* extract was able to significantly suppress the influence of HGF in a TNBC cell line. The anti-metastatic properties were governed by the ability of BFE to reduce HGF/c-Met signalling events through suppression of c-Met tyrosine kinase receptor phosphorylation. The true clinical value of BFE as a therapeutic modality requires substantial work; however, BFE may demonstrate the potential to be used as an adjuvant therapy in the clinical management of breast cancer metastasis in TNBC-classified patients with poor prognosis.

## Abbreviations

AKT: protein kinase B
BFE: *Boswellia frereana* extract
c-MET: hepatocyte growth factor receptor
ECM: extra cellular matrix
GC–MS: gas chromatography–mass spectrometry
HGF: hepatocyte growth factor
MAPK: mitogen-activated protein kinase
NF-κB: nuclear factor-κB
PI3K: phosphoinositide 3-kinase protein kinase B
STAT: signal transducer and activator of transcription proteins
TNBC: triple negative breast cancer
